# Biography—Dr. Baillie

**Published:** 1823-12-01

**Authors:** 


### XIII.

### BIOGRAPHY.—(Dr. BAILLIE.)

"—————— tua splendida facta
"Nulla unquam ex animis tollent oblivia nostris."

It is with sincere sorrow we have to record the death of one of our ablest physicians and best men! In Dr. Baillie, the profession has lost a father and a friend! When the mind of the medical practitioner was harrassed with doubt, and that of the patient with despondency, the advice of this amiable and experienced physician was eagerly sought by both parties. To Dr. Baillie's honour, the practitioner might safely confide his reputation—to his judgment, the patient might implicitly confide his life. There is one great and fundamental principle in medical ethics from which Dr. Baillie never deviated—that of doing as much good as possible to the patient, with as little injury as possible to those previously in attendance. Although we firmly believe that there is, at this moment, more honorable feeling in operation among the different orders and individuals of the faculty than at any former period, notwithstanding the clashings of interest, and the generally overstocked state of the profession, yet it is not to be disguised that there are too many who are ready to embrace every opportunity of enhancing their own influence or popularity, at the expence of those who go before or co-operate with them. This is a great breach of good faith—and is often most injurious in effect, when least tangible in form. Dr. Baillie was not the man, who by word, look, or action, would cast the slightest shadow of reflection on the conduct or opinion of the general practitioner or the ordinary physician in attendance. If he differed in opinion, or disapproved of the measures of others, that difference or disapprobation never appeared beyond the precincts of the consultation room. He knew too well the uncertainty of medical evidence, and the proneness of the human mind to error, not to view with tenderness the failings or even the indiscretions of others. In aiding the former or reproving the latter, he alarmed not the patient, and offended not the practitioner.* That such conduct was founded on the wisest worldly policy in the end, as well as on the noblest moral

* That this delicacy or lenity might, in some instances, have the effect of screening delinquency, palliating error, or veiling ignorance, we are not prepared to deny. Indeed we have heard it asserted that such was actually the case, and that Dr. Baillie leaned sometimes so far on the side of mercy as to infringe on the sacred pale of justice. If this were the fact, of which, by the bye, we never heard an authenticated instance, how much more amiable is the failing than the opposite extreme, where stern justice is never tempered with heaven-born mercy!

principle at the beginning, will be acknowledged by most men who have narrowly watched the career of physicians. That reputation *alone* can be solid or lasting which is based on the adjudication of the only legitimate tribunal—the voice of the profession ; and he who hopes to raise it on any other foundation, or imitates the example of those who use unworthy means to attain popular fame or worldly pelf, will find himself wofully deceived in the end. The instances to the contrary are but exceptions to general rules, and ought, in fact, to stand as land-marks against hidden dangers. We hold, it therefore, as certain that, while Dr. Baillie's death must prove a public loss, the example of his professional life will long operate with beneficial influence on those members of the profession who remain behind.

"Hoc vigeant mandata, nec ulla obliteret ætas."

Dr. Baillie's character as a physician, is too well known to need much comment here. He has been considered by many as timid in practice, undecided in prognosis, and sceptical in the powers of medicine. But the mind as well as the body of man is moulded by the circumstances in which he is placed ; and he who reflects on the station which the deceased has long held in medical society cannot wonder that the abovementioned traits of professional character should become somewhat conspicuous without any implication of the talents or judgement of the individual. Every one is aware that for many years past, Dr. Baillie was rarely called in, except in chronic, or the advanced stages of acute diseases. Is it not evident that, under such circumstances, he had far more frequently to deal with intractable and fatal cases, than with those of an opposite description. Hence arose an habitual caution, both in treatment and prognosis, which might readily be mistaken by the casual observer for timidity or indecision.

Dr. Baillie's early and intimate knowledge of anatomy, his careful observation of phenomena, and his long and ample experience, joined perhaps to a natural tact, enabled him to acquire a very considerable facility in forming a just estimate of the nature, seat, and extent of a disease—and it was in this facility or sound judgement, in our humble opinion, that Dr. Baillie's principal forte lay. Knowledge is power—and this power was, of all others, the most serviceable to Dr. Baillie, as it is indeed to every consulting physician, who can have little control over the daily management of diseases, but who may be of infinite service both to patient and ordinary practitioner, by aiding in the institution of correct views, and sound general principles.

As far then as chronic diseases were concerned, we conceive that the charge of timidity against Dr. Baillie was unfounded, and in some instances of violent and acute diseases, where we acted in conjunction with this lamented physician, we saw no want of decision, but a ready co-operation or acquiescence in the most vigorous measures.

In consultation with his brethren, Dr. Baillie was affable, candid, and free from the slightest tendency to overbearing, even with the humblest members of the profession—a trait of character which it would be well for some of the higher orders to imitate. Nothing, in fact, can be more indicative of a weak mind and defect of real knowledge than a dogmatical and domineering conduct in consultation. From the situation which Dr. Baillie so long held, as a kind of oracle in medicine, it was necessary for him to be extremely guarded in his diagnostic or prognostic conversations with the patient or friends, since every word he said was sure to be well remembered afterwards. On this account he was forced to give Delphic responses, in general, which might admit of different interpretations according as the event turned out. Nor was he to be blamed for this :—he well knew that no physician, had he even a thousand years' experience, can give positive predictions in medicine, unless he runs the risk of being more frequently wrong than right—yet predictions are always unreasonably expected by patients or friends, and they must be met by mystery oftener than by candour. Where there was no doubt, however, in his own mind, respecting the event, whether favourable or fatal, Dr. Baillie very properly gave candid and honest opinions either to the patient or friends.

Dr. Baillie, as might be expected, used very simple prescriptions, and seldom ventured on new or hazardous remedies until they were long employed by others, well knowing that not one in twenty of them would stand the test of experience, and his patients were not of that class on which experiments could be safely tried. He was well aware also that the great object is to ascertain the seat and nature of the disease, and the proper indication to pursue—the *means* are generally found among our most common and safe remedies.

We have no reason to believe that Dr. Baillie was an avaricious physician; but his patients, for very many years before his death, were in that rank of life that rendered generosity unnecessary—perhaps injurious. We have, no doubt that, where this amiable physician saw reason for thinking the fee could ill be spared, he invariably declined it. One thing is certain, that his visits and advice were always at the service of his professional brethren in the hour of illness—even when the act of kindness interfered with his regular professional pursuits. In this, it is true, he was not singular; for to the credit of the profession be it recorded, that nine in ten of its members go more willingly to assist each other in sickness than to those patients who are to pay for their professional attendance.

Considering that Dr. Baillie dedicated a good many of his early years to pathological researches, and was the author of an esteemed work on Morbid Anatomy, it is somewhat remarkable that he did not evince so much regard to that minute pathology which, for many years past, has been so successfully cultivated, as was to be wished or expected. We know indeed that our ardour in most pursuits (excepting perhaps that of wealth) abates as we decline in the vale of years; but Dr. Baillie had not arrived at such an advanced age as to account for this lukewarmness, on the above principle. He probably thought that minute pathology was much less important than careful observation of symptoms—but assuredly it is only by a combination of the two, that medical science can ever be materially advanced. We conceive that a knowledge of minute structure, both sound and morbid, is just as necessary to the physician as to the surgeon—for without this knowledge, he cannot trace the effects of diseases, nor connect these effects with their correspondent symptoms.

Whether Dr. Baillie was deeply versed in medical literature, ancient and modern, it is difficult to say, as he very rarely alluded to one or the other in his conversation or writings. The probability is that he did not attach much importance to ancient records, and that he preferred the book of nature before him, rather than the imperfect, confused, and too often false speculations of his predecessors. In this respect he had the example of his illustrious relative, John Hunter, before his eyes. After all, what is it that we prize in the writings of the ancients?—only those passages which our own experience approves.

The writings of Dr. Baillie are characterized rather by careful observation than expanded views. His great work on morbid anatomy, though it exhibits accurate representations and concise descriptions of the more common diseased structures, is, at the same time, highly defective in the histories and symptoms connected with them. It is indeed more than probable that most of the drawings and descriptions were taken from morbid appearances presented in the dissecting room, or in a public hospital, where of course the living phenomena, or, at least, the early history, could not be well ascertained. The work itself was published before he got into the great stream of private practice, where opportunities of connecting symptoms with changes of structure were every where at hand.

In private life we have reason to believe that Dr. Baillie was as amiable as in his public capacity. It is well known that his purse was the first open for all charitable institutions, and for many years before his death he drew, at stated periods, round his hospitable table, a succession of all the more distinguished of his brethren, (without regard to rank,) for the promotion of friendly intercourse and professional conversation. At these assemblages of talent and information Dr. Baillie always enjoyed the "feast of reason and the flow of soul," as much as the youngest of the company—inspiring them with that beneficence of character and conduct for which he was himself so distinguished. Long may the example of this great and good man continue to operate on the upper as well as the lower classes of medical society!

Since writing the above, (which was sketched while wandering on the banks of the Rhine,) we have seen Mr. Bell's biographical notice of Dr. Baillie, as delivered to his pupils in an introductory lecture in the school originally adorned by the deceased.

Dr. Baillie was the son of the Rev. James Baillie, Professor of Divinity in the University of Glasgow. His mother was the sister of Dr. William and Mr. John Hunter. He took his degree in Baliol College, Oxford, and finally came under the superintendence of his uncle, Dr. William Hunter, who brought him forward in life, and got him appointed physician to St. George's Hospital. Dr. Hunter wished Dr. Baillie to succeed him; and for that purpose associated with him his assistant, Mr. Cruickshanks. At his death he also bequeathed to Dr. Baillie the use of his anatomical preparations for 30 years. His prelections were remarkable for that lucidus ordo and clearness of expression resulting from a perfect conception of the subject. They were conveyed to his pupils in the simplest and most intelligible way. It appears from Mr. Bell that " his association in the lectureship made his duties (in Windmill Street) very unpleasant to him," otherwise he would have continued much longer his course of instruction to professional youths.

This amiable physician has presented his books, plates, and specimens of morbid anatomy to the College of Physicians, together with a sum of money to be expended in keeping them in order. A handsome tribute to the memory of Dr. Baillie was inserted in the College annals, on the 30th September, from which we shall extract a single sentence. " To the rising generation of physicians it may be useful to hold up, for an example, his remarkable simplicity of heart, his strict and clear integrity, his generosity, and that religious principle by which his conduct seemed always to be governed, as well calculated to secure to them the respect and good will of their colleagues and the profession at large, and the high estimation and confidence of the public."

Mr. Bell well observes that " we cannot estimate too highly the influence of Dr. Baillie's character on the profession to which he belonged. All wished to imitate his life—none to detract from his fame. Every young physician who hoped for success, sought his counsel:—and I have heard him forcibly represent the necessity of a blameless life, and that, unless medical reputation be joined with purity of private character, it neither could be great nor lasting."

Dr. Baillie was only 63 years of age, though he appeared to be more than that in constitution. Early in the spring of this year he became affected with the influenza then prevailing, and he did not suffer himself to remain quiet at home, but continued to visit many patients who were not so ill as himself. We were present at the last medical dinner which he gave, in the beginning of the present summer. We all remarked the formidable change in his appearance. He was constantly coughing and expectorating; but seemed to think there was nothing more than a trifling affection of the mucous membrane of the lungs. The complaint, however, was producing rapid emaciation, and ultimately carried him off.

Dr. Baillie has left a wife, a son, a daughter, and two sisters, to deplore his loss. He must have realized, after all expences, a sum of more than one hundred thousand pounds.

And now that the last debt of Nature has been paid by Dr. Baillie, it will be silently asked by many a mind—" who is to take his place?" We think we may safely affirm, without wounding the self-love or the vanity of any man, that no individual will ever again hold that place in the public and the professional estimation which was so long occupied by the deceased. Not that there is any lack of talent to supply the vacancy, but because there is now too much information expanded among a great number ever to permit any individual to become that kind of monarch in medicine which Dr. Baillie was. The last 25 or 30 years have effected a great revolution in this respect. The unexampled activity of the human intellect—the rapid and wide diffusion of knowledge—the boundless zeal in exploring the laws of the animal economy, in health and in disease—and the improvement in the education of the general practitioner, are rapidly bringing every man to his proper level, and almost threaten to bring all men to the same level! There never can again be seen a monarchy, nor even an aristocracy in medicine. A fungous and temporary reputation among nurses, old women, hypochondriacs, and fashionables, will indeed every year spring up and decay; but any thing like a great ascendency in professional estimation can only result from the birth of some genius like that of a Hunter, which is not to be expected above once in a century. Inequalities of talent or capacity will ever indeed be found among men; and allowing knowledge to be equal, some men will always be able to apply it with more advantage and effect than others; but beyond this, there will soon be little else than a general democracy in medical science—at least among the more zealous and distinguished members of the profession.

